# Genome Sequence of *Elizabethkingia meningoseptica* EM1, Isolated from a Patient with a Bloodstream Infection

**DOI:** 10.1128/genomeA.01137-16

**Published:** 2016-10-27

**Authors:** Shicheng Chen, Marty Soehnlen, Edward D. Walker

**Affiliations:** a Department of Microbiology and Molecular Genetics, Michigan State University, East Lansing, Michigan, USA; b Michigan Department of Health and Human Services, Bureau of Laboratories, Lansing, Michigan, USA

## Abstract

*Elizabethkingia meningoseptica* EM1 was isolated from a whole-blood sample from a female patient. The draft genome sequence of Em1 contains 4,038,467 bp, with a G+C content of 36.37%. A preliminary genome analysis showed that Em1 contains genes conferring resistance to β-lactams. The bacterium has hemolysin genes and a set of genes involved in heme uptake and heme utilization, showing its potential to cause bloodstream infections. A clustered regularly interspaced short palindromic repeat (CRISPR)/CRISPR-associated protein (CRISPR/Cas) system was identified. Average nucleotide identity (ANI) analysis assigned the bacterium to the species *E. meningoseptica* (ANI, >95%)*.* The annotated genome sequence provides the genetic basis for revealing its role as a pathogen in humans.

## GENOME ANNOUNCEMENT

Bacteria of the genus *Elizabethkingia*, belonging to the family *Flavobacteriaceae*, are strictly aerobic, nonmotile, nonfermenting, non-spore-forming, and yellowish Gram-negative rods (1, 2). Four species were assigned to this genus, *Elizabethkingia meningoseptica* (3), *Elizabethkingia anophelis* (4), *Elizabethkingia miricola* (3), and *Elizabethkingia endophytica* (5), with the first three considered to be medically important (6). *E. meningoseptica* (epithet name referring to the association of this bacterium with both meningitis and septicemia) was first described as a type species and is the best studied (2). Like other bacteria distributed in clinical settings, *Elizabethkingia* species have the ability to survive and proliferate under nutrient-poor conditions and to be resistant to disinfection reagents (7). The nosocomial infections caused by *E. meningoseptica* cause a high mortality rate (>20%) in immunocompromised adult patients with underlying health conditions, or in premature infants and newborns (8). In this paper, we report the genome sequence of *E. meningoseptica* strain Em1, isolated from a patient with a bloodstream infection.

The draft genome was sequenced by Illumina MiSeq paired-end sequencing technology at the Research Technology Support Facility (RTSF) at Michigan State University. The reads were assembled using SPAdes (version 3.9.0) and further edited using DNAStar (version 1.12). After removing short contigs and sequences with potential contamination, it resulted in 10 contigs with an *N*_50_ of 988,765 bp. The maximum contig size was 1,083,713 bp. The assembled genome comprises 4,038,467-bp nucleotides with a G+C content of 36.37%. Gene annotation was carried out by NCBI Prokaryotic Genome Annotation Pipeline (PGAP, 3.3 [http://www.ncbi.nlm.nih.gov/genome/annotation_prok/]). A total of 3,656 genes yielded a coding capacity of 4.0-M nucleotides. There were at least 55 RNA sequences, including 45 tRNAs, seven rRNAs, and three noncoding RNAs (ncRNAs) in the genome.

Many genes encoding proteins involved in virulence, disease, and defense were identified in the genome. We also found protein-coding genes related to capsule formation, which possibly enhances the ability of the bacteria to cause infection, escape from phagocytosis, and attach to the surfaces of medical devices. Several putative hemolysin, heme uptake, and heme utilization genes involved in breaking down red blood cells were annotated, indicating that EM1 was equipped to be an opportunistic pathogen in bloodstream infections (7). More than two dozen genes conferring resistance to β-lactams (13 β-lactamases, 5 metallo-β-lactamases [MBLs], and four penicillin-binding proteins) were annotated in the EM1 genome. Isoprenoid biosynthesis genes essential for cell survival and immune stimulation in hosts exist in this bacterium (9). A battery of genes encoding the CRISPR/Cas system were found (10).

The 16S rRNA gene in Em1 showed 99%, 99%, 98%, and 98% identity to those in *E. meningoseptica* ATCC 13253, *E. anophelis* NUHP1, *E. miricola* ATCC 33958, and *E. endophytica* JM-87, indicating that the method based on 16S rRNA sequencing cannot offer a sufficiently high resolution for comparison. Average nucleotide identity (ANI) analysis using *E. meningoseptica* ATCC 13253 (accession no. NZ_BARD00000000.1), *E. anophelis* NUHP1 (accession no. NZ_CP007547), and *E. miricola* ATCC 33958 (accession no. NZ_JRFN01000000) as reference genomes showed average nucleotide identities of 98.61%, 81.43%, and 81.77%, respectively, demonstrating that EM1 belongs to the species *E. meningoseptica* (ANI, >95%).

### Accession number(s).

This whole-genome shotgun project has been deposited at DDBJ/ENA/GenBank under the accession no. MCJH00000000. The version described in this paper is version MCJH01000000. The BioProject designation for this project is PRJNA336273. The BioSample accession number is SAMN05507161.
